# Sodium selenite/selenopeptide chitosan antioxidant film for postharvest preservation of grape

**DOI:** 10.1016/j.fochx.2026.103785

**Published:** 2026-03-23

**Authors:** Li Li, Shushu Pu, Zhixuan Fan, Yaping Liu, Jianbing Di, Yu Wang, Wei Ji

**Affiliations:** a College of Food Science and Engineering, Shanxi Agricultural University, Shanxi Taigu 030801, China; b Shanxi Province Innovation Center for Storage and Processing Technology of Fruit and Vegetable, Shanxi Taigu 030801, China; c College of Horticulture, Shanxi Agricultural University, Shanxi Taigu 030801, China

**Keywords:** Chitosan film, Selenopeptide, Sodium selenite, Antioxidant properties, Deep eutectic solvent, Grape preservation

## Abstract

Chitosan is an excellent alternative to plastic, a natural polymer that is non-toxic, biodegradable, and biocompatible. However, chitosan film exhibits relatively poor mechanical properties. Therefore, the films were successfully modified by deep eutectic solvents, and the effects and mechanisms of sodium selenite and selenopeptides, at concentrations of 0.25% and 0.4%, on the characteristics of chitosan films were comprehensively studied. The evaluation of film properties was supported by Fourier transform infrared spectroscopy, X-ray diffraction, and scanning electron microscopy.Significant improvements in the films' UV absorption and shielding abilities were observed by incorporating sodium selenite and selenopeptides. The addition of sodium selenite and selenopeptides significantly enhanced the antioxidant and antibacterial properties.The combination of chitosan with sodium selenite and selenopeptides provides a strategy for simultaneously reinforcing mechanical, UV-protective, antioxidant, and antimicrobial characteristics. Such multifunctionality supports the potential expansion of chitosan films into advanced packaging applications.

## Highlights

• Chitosan-based films incorporated with sodium selenite and selenopeptides were successfully developed and characterized.
• The DCS exhibit superior antioxidant and antibacterial activities than chitosan.
• Our films possess enhanced UV-blocking ability and mechanical performance.
• DCS films can effectively extend the shelf life of grapes.

## 1 Introduction

Green packaging has attracted significant interest as a crucial component of lowering environmental pollution and promoting sustainable development in light of the current state of the world's environment, which is becoming more and more deteriorated, and the growing consciousness of environmental protection (Edo et al., 2025). Plastic food packaging materials primarily comprise high-molecular-weight polymers, supplemented with functional additives such as plasticizers, stabilizers, and antioxidants. These materials undergo processing and forming under appropriate temperature and pressure conditions. Common plastic packaging materials include polyethylene, polypropylene, and polyethylene terephthalate, among others. The widespread utilization of plastic packaging materials in food packaging can affect the flavor characteristics of food products (Bai et al., 2024). Furthermore, this type of material is not biodegradable, leading to significant environmental white pollution. Moreover, when it comes into contact with food, specific harmful compounds, including bisphenol A and phthalates, along with other potentially dangerous chemicals within the material, may migrate into the food via processes such as diffusion, desorption, and adsorption, thereby posing potential safety risks to human health (Etxabide et al., 2022). Such constraints emphasize the critical necessity for the development of biodegradable alternative materials to advance sustainable food packaging solutions.

Chitosan (CS), a basic cationic polymer composed of 2-amino-2-deoxy- (1, 4)-β-D-glucosamine units, is produced by deacetylating chitin (Shete et al., 2024). CS possesses a wide range of remarkable properties. It is inherently non-toxic, environmentally biodegradable, and exhibits excellent film-forming capability. In addition, CS exhibits outstanding biocompatibility and functional versatility, which have led to its extensive use in food packaging, biomedical devices, and pharmaceutical formulations (Zidan et al., 2023). Despite its many advantages, CS exhibits limited antioxidant activity and poor mechanical performance, which highlights the need for improvement. As a result, its ability to carry out its preservation function is constrained.

CS has poor flexibility after film formation, which can be improved by adding plasticizers. Deep eutectic solvents (DESs) represent a type of sustainable solvent system generated through the precise combination of hydrogen bond donors and acceptors at a predetermined molar ratio, offering advantages such as low toxicity, biodegradability, simple preparation, and excellent solvation ability. Since DESs and ionic liquids (ILs) share many traits and attributes, DESs have been generally recognized as a novel category of ILs. In 2016, the initial hot-pressing of CS films employed choline chloride (ChCl) and citric acid as plasticizing agents (Galvis-Sánchez et al., 2016). DES films exhibit greater flexibility, opacity, and water vapor transmission than films containing only citric acid. The integration of DES into CS can elevate their ultimate ductility, thereby boosting the mechanical properties of CS films (Jakubowska et al., 2020). DES can reduce the tensile strength, rigidity, fluidity, and electrostatic potential of CS film, thereby enhancing their plasticity (Jakubowska et al., 2021). As innovative solvents, DESs can address the challenges associated with chitin extraction as well as the inherent brittleness and limited ductility of CS films. The incorporation of DESs significantly improves the antioxidative ability of polymeric films (Mercadal et al., 2024).

Selenium exists as inorganic or organic species, with the former exhibiting greater toxicity and lower bioavailability (Souza et al., 2022). Organic selenium primarily exists as selenoamino acids, particularly selenomethionine and selenocysteine, which are the primary biologically active forms in plants and animals (Shahidin et al., 2025). Organic selenium is less harmful to the host and lasts longer in both humans and animals. Additionally, organic selenium is safer, more bioavailable, and more biologically active (Hadrup & Ravn-Haren, 2021). The antibiotic susceptibility of *Pseudomonas aeruginosa* was increased and its pathogenicity reduced following treatment with sodium selenite (SSE), which triggered oxidative stress and interfered with the bacterium's quorum-sensing pathways (Kong et al., 2021). SSE also enhances the antioxidant capacity of cells through various mechanisms. Egg yolk selenopeptides (SeP) enhanced in vitro antioxidant activity (Zhao et al., 2024). Yeast peptides enriched with selenium, comprising selenium and bioactive constituents, can act as antioxidants. They can be applied either as dietary supplements to enhance the body's systemic antioxidant defenses or as cosmeceutical formulations to mitigate oxidative damage in skin tissues (Guo et al., 2020). The administration of organic selenium forms induced enhanced immune responses. In addition, it led to elevated selenium levels in tissues compared to inorganic forms (Zhang et al., 2020). Nevertheless, the synergistic potential of SSE and SeP incorporated into a DES-plasticized CS system, which may provide increased bioactivity and decreased cytotoxicity, has not yet been investigated in the literature.

Based on the description above, this work used a mixture of CS, SSE, SeP, and DES to fabricate extremely multifunctional films. The effects of SSE or SeP content on the thermal stability, moisture resistance, mechanical properties, and water barrier performance of blend films were systematically evaluated. The findings offer practical observations for the development of biodegradable films.

## 2 Materials and methods

### 2.1 Materials

CS (≥95% deacetylated, viscosity: 100–200 mPa·s) was obtained from Shanghai Macklin Biochemical Co., Ltd. SSE and citric acid were purchased from Tianjin Damao Chemical Reagent Factory. SeP, possessing a selenium concentration of at least 2000 mg/kg, was purchased from Bai Chuan Kangze Biotechnology Co., Ltd. Choline chloride was obtained from Beijing Solarbio Science & Technology Co., Ltd.

### 2.2 Preparation of composite films

The DES was prepared by mixing ChCl with citric acid (1:1). The mixtures underwent continuous agitation for a duration of 60 min, at a constant temperature of 80 °C until the formation of a transparent solution. To begin with, 2% (w/v) CS was dissolved in an acetic acid solution (1% v/v), subjected to magnetic stirring at 800 rpm and 40 °C over a 6-h period. Subsequently, DES was added at a proportion of 30% (w/w) relative to CS and mixed thoroughly via mechanical stirring. Next, 2.5 mg and 4 mg of SSE, as well as SeP, were incorporated into the solution above. Following preparation, the mixture was continuously stirred for 2 h before being poured into an acrylic glass dish (20 cm × 20 cm × 5 mm). The solution was allowed to dry at 25 °C, subsequently conditioned at 25 °C and 55% RH for 48 h to obtain the films. The resulting films, prepared with SSE (2.5 mg for DCS-1 and 4 mg for DCS-2) and SeP (2.5 mg for DCS-3 and 4 mg for DCS-4), were designated as CS, DC, DCS-1, DCS-2, DCS-3, and DCS-4, respectively.

### 2.3 Structure characterization of composite films

The film's microstructure was examined with a field emission scanning electron microscope (FE-SEM, TESCAN MIRA3, Czech Republic). Attenuated total reflectance (ATR) mode was employed for FTIR analysis on a Tensor 27 spectrometer (Bruker, Germany), spanning a spectral range from 4000 to 400 cm^−1^, at a resolution of 4 cm^−1^, with 32 scans conducted for each sample. X-ray diffraction (XRD) was employed to investigate the chemical construction of the films, employing a diffractometer manufactured by Dandong Haoyuan Instrument Co., Ltd. (China), functioning at 40 kV and 40 mA.

### 2.4 Water barrier performance

#### 2.4.1 Water properties

The water properties were evaluated employing the following procedure. Samples were sectioned into 20 mm × 20 mm pieces. Their initial mass was recorded (W_0_), and desiccated in a drying chamber maintained at 105 °C for 24 h, followed by being transferred to a desiccator to achieve a stable mass before re-weighing (W_1_). Subsequently, the desiccated films were immersed. Then the film was positioned within a thermostatic incubator for a 24-h interval. The undissolved CS films were dried in a heated chamber. And the films were weighed after maintaining for a period until no further weight loss occurred (W_2_) (Zhang, Jiang, et al., 2022). The pre-weighed (W_1_) film samples were immersed for 24 h, and then the external moisture on the film was removed with absorbent paper and weighed again (W_3_) (Tan et al., 2019). The film's water performance was determined in accordance with the subsequent equations:

(1) $$\text{Water content}\;\left(\mathrm{WC}\right)\;\left(\%\right)=\left(W_{0}-W_{1}\right)/W_{0}\times 100$$

(2) $$\text{Water solubility}\;\left(\mathrm{WS}\right)\;\left(\%\right)=\left(W_{1}-W_{2}\right)/W_{1}\times 100$$

(3) $$\text{Swelling degree}\;\left(\mathrm{SD}\right)\;\left(\%\right)=\left(W_{3}-W_{1}\right)/W_{1}\times 100$$

#### 2.4.2 Water vapor transmission rate (WVTR)

The WVTR of the plastic films was determined in accordance with GB/T 21529–2008 using the gravimetric (cup) method. Test specimens were hermetically sealed onto the openings of permeation cups filled with either anhydrous calcium chloride desiccant (for moisture barrier evaluation) or distilled water (for moisture absorption analysis). The assembled cups were put into a chamber (Jinan Thinker WVTR-920-3S) maintained at (38 ± 0.6)°C and (90 ± 2)% relative humidity, with forced air circulation at ≥0.5 m/s. Mass measurements were recorded gravimetrically at 24-h intervals to quantify vapor permeation. The WVTR was calculated as the steady-state mass change per unit area per 24-h period, expressed in g/ (m^2^·24h).

#### 2.4.3 Contact angle

To assess the wetting characteristics of the film surface, the dynamic contact angle (CA) method was used to determine the CA of the samples. The film was set flat on a slide and put on a contact angle meter's sample stage (CA-100 Contact Angle Meter, China). The test was conducted using a 5 μL droplet as the test liquid. Immediately after the droplet made complete contact with the sample surface, initiate the measurement sequence and record the left and right CAs within 20 s.

### 2.5 Physical properties

#### 2.5.1 Appearance and optical properties

To assess the films' UV absorption and shielding capabilities, Specimens of the films, each measuring 10 mm × 50 mm, were meticulously affixed to the interior wall of a quartz cuvette before analysis. Samples were placed in a quartz cuvette. UV–Vis spectra for transmittance and absorbance of the films were acquired utilizing an Agilent Cary 60 spectrophotometer across the 200–800 nm wavelength spectrum, employing a blank quartz cuvette for baseline adjustment.

#### 2.5.2 Mechanical properties

Mechanical properties of 70 mm × 10 mm films were tested using a tensile machine (Model 5544, Instron, Norwood, MA, USA). The properties were tested under controlled conditions, employing a tensile speed of 5 mm/min up to the point of fracture.

### 2.6 Antioxidant ability

In a test tube, the sample solution was mixed with the ABTS^+^ solution (1,4) using oscillation (Zhao, Huang, et al., 2025). After that, the combination was shielded from the light. The solution was to stand for six minutes. Absorbance values at 734 nm were quantified employing a UV–Vis spectrophotometer (Agilent Cary 60). The reaction mixture solution was prepared by combining the DPPH· ethanol solution and the film solution (4,1). The solution was subsequently allowed to react for 30 min. The sample was measured at a 517 nm wavelength. Using Eq. (4), the DPPH· scavenging rate was quantified.

(4) $${\text{ABTS}}^{+}/\text{DPPH}\cdot \text{scavenging activity}\;\left(\%\right)=\left(A_{0}-A_{1}\right)/A_{0}\times 100$$

A_0_ and A_1_ corresponded to the absorbance of the control group and the tested films, respectively.

### 2.7 Antibacterial ability

The antibacterial activity of the films against *Escherichia coli* (ATCC 25922) and *Staphylococcus aureus* (ATCC 25923) was assessed using the agar diffusion assay. Before testing, bacterial cultures were cultivated in Luria–Bertani (LB) broth at 37 °C for 18–24 h under agitation at 150 rpm, achieving an approximate cell density of 10^7^ CFU/mL. Subsequently, the sterile nutrient agar (NA)’s surface was evenly coated with bacterial culture. Using sterile tweezers, a sterilized perforator (8 mm in diameter) was then positioned onto the agar surface. Finally, an aliquot of 100 μL solution was dispensed into the well before incubation at 37 °C for 14 h.

### 2.8 Grape preservation

Mature grapes of similar dimensions and ripeness were acquired from a nearby supermarket located in Taigu, China. Following disinfection with 70% ethanol, the grapes were systematically allocated to plastic containers, wherein each cohort consisted of 10 specimens, and subsequently enclosed with CS, DC, DCS-1, DCS-2, DCS-3, or DCS-4 films. All groups were kept at ambient temperature (25 °C), with unpackaged grapes serving as the control. Weight loss (%) and firmness (N) were measured over a 9-day storage period, following methods described in prior studies (Zheng et al., 2023).

### 2.9 Statistical analysis

The experiments were replicated thrice, with all outcomes expressed as mean ± standard deviation (SD). One-way analysis of variance (ANOVA) was conducted to evaluate statistically significant disparities (*p* < 0.05), followed by Tukey's multiple range test for subsequent pairwise assessments.

## 3 Results and discussion

### 3.1 Characterization

#### 3.1.1 SEM

**Fig. 1:**
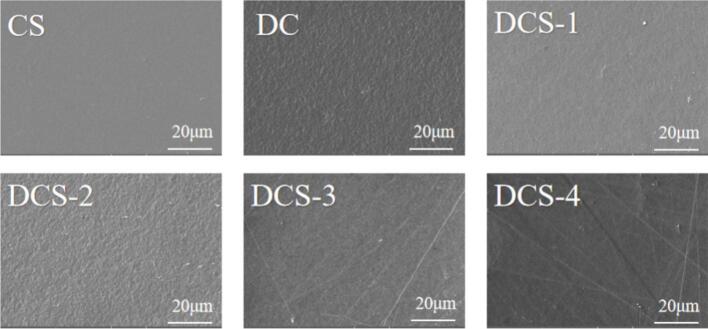
SEM photo of the surface of films.

The film images seen in Fig. 1 do not exhibit any obvious phase differences. There was no discernible phase separation in the SEM photos. The CS film had a smooth and dense microstructure, showing a cohesive structure between CS molecules. High similarity structure of the DCS films was shown to resemble the CS film, as confirmed by SEM analysis. Additionally, when SeP and SSE were added into the CS film, the film displayed minor irregularities and a slight reduction in uniformity, which are often indicative of a high degree of compatibility between both substrates. Consistent findings were reported for film reinforced with Ag/ZnO nanoparticles (Nguyen et al., 2025). The films' surface containing SeP and SSE exhibited no visible cracks or pores, indicating strong compatibility between the particles and the film matrix, thereby meeting the criteria for effective active food packaging.

#### 3.1.2 FTIR

**Fig. 2:**
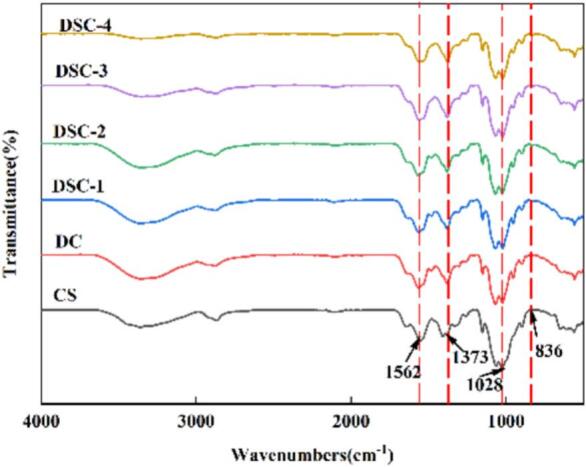
FTIR spectra of the films.

FTIR was conducted in order to analyze their molecular interactions. Fig. 2 displays the FTIR spectra of CS, DC, DSC-1, DSC-2, DSC-3, and DSC-4 films. DSC displays distinct absorption peaks at 3366 cm^−1^, 1652 cm^−1^, 1562 cm^−1^, and 1373 cm^−1^, due to the vibrations of —OH and —NH, stretching vibration of C=O in amide I, deformation vibration of N—H, and stretching vibration of —C—N in amide II and amide III, respectively. Peaks were at 2880 cm^−1^ and 1639 cm^−1^ within the spectra of the CS film. These were attributed to the stretching vibrations of —CH and C—O groups within the amide I band, respectively. By contrast, no additional peaks were detected in the DSC films compared to those lacking Se, suggesting that no new chemical bonds formed between Se and the film matrix. The spectra of Se-containing films closely resembled those observed in pristine CS films. The FTIR peaks of the films were revealed at approximately 3366 cm^−1^. As the Se content increased, these peaks shifted, which implies the occurrence of hydrogen bond formation (Smirnov et al., 2021). In summary, the findings above suggest that Se could potentially form hydrogen bonds with CS and DES. The establishment of hydrogen bonds can enhance the mechanical characteristics and thermal resilience of the films (Zhang, Chen, et al., 2022).

#### 3.1.3 XRD

**Fig. 3:**
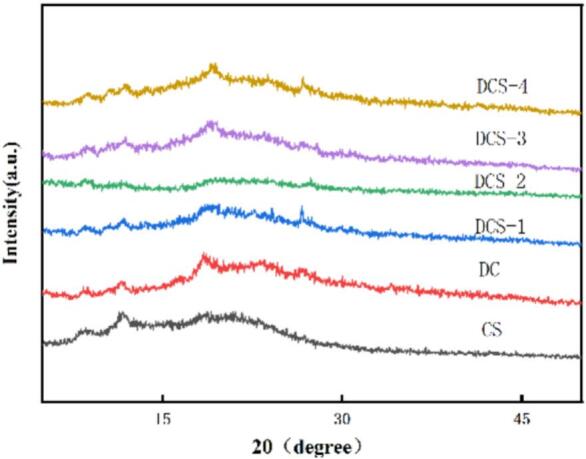
XRD spectra of the films.

Fig. 3 illustrates that no distinct sharp peaks were observed, with the XRD patterns of the six film groups displaying notable similarity. The DCS sample exhibits two diffraction peaks centered at 2θ values of 11.75° and 19.36°. Neat CS exhibited two peaks at 11.49° and 19.23° representing its amorphous structure as supported by previous studies (Parveen et al., 2024). Upon incorporation of SSE, the XRD patterns of DCS films exhibit a transition from sharp to broader or flattened peaks, indicating a significant reduction in crystallinity. This peak broadening is ascribed to the weakened intermolecular interactions within the CS film, primarily due to the disruption of hydrogen bonding and crystalline arrangement caused by the additive. Concurrently, when the SSE amount raised, the intense diffraction peaks of SSE diminish significantly or disappear entirely, suggesting that SSE molecules are effectively “encapsulated” or “dispersed” within the CS film, preventing the formation of large-scale ordered crystalline lattices (Thanh Huong et al., 2023). When the SSE amount raised, the diffraction peak intensity near 19° diminished. This finding provides strong evidence for the weakening of the hydrogen-bond interaction network in the film. This observation strongly supports the disruption of the intermolecular hydrogen bond network within the film (Zhao, Liu, et al., 2025). And new interactions between CS and SSE may be forming and becoming more pronounced (Yin et al., 2023). With the addition of SeP, the XRD peak near 19° in CS films slightly shifts to higher angles, indicating altered lattice spacing and the formation of new ordered structures due to reorganized intermolecular interactions between SeP and CS. As SeP content rises, the diffraction peak intensity around 20° progressively weakens, confirming strengthened intermolecular interactions within the matrix (Li et al., 2023).

### 3.2 Water barrier performance

#### 3.2.1 WC, WS, SD

The thickness, WC, WS, and SD of films were measured. As illustrated in Table 1, the inclusion of SSE and SeP in the film formulation caused a notable rise in film thickness, rising from approximately 0.018 mm to approximately 0.045 mm. We found that the films' WC dropped from 29.83% to 16.66%. The development of hydrogen bonds between SeP, SSE, CS, and DES most likely caused this decline by reducing the quantity of —OH that could interact with water molecules (Hao et al., 2025). CS is a hygroscopic polysaccharide. As illustrated in Table 1, the WS of the DCS-4 film was 47.22%, demonstrating an upward trend with the incorporation of SeP. This increase can be attributed to SeP's inherent hydrophilicity. Besides, a significant rise in SD with SeP and SSE was recorded. This occurrence can be explained by the existence of hydrophilic groups, for example, amino groups, in the SeP molecules. These groups enhance the overall hydrophilicity of the membrane, facilitating stronger interactions with water molecules in an aqueous environment, thereby leading to an elevated SD.

**Table 1:** Water barrier performance of the films.

Sample	Thickness (mm)	WC (%)	WS (%)	SD (%)
CS	0.018 ± 0.002^c^	29.83 ± 1.08^a^	22.71 ± 0.32^f^	46.97 ± 0.87^e^
DC	0.038 ± 0.002^b^	20.28 ± 0.48^b^	25.26 ± 0.46^e^	50.35 ± 0.61^d^
DCS-1	0.042 ± 0.006^ab^	17.75 ± 0.17^c^	28.42 ± 0.25^d^	53.9 ± 0.98^c^
DCS-2	0.046 ± 0.002^a^	16.64 ± 0.62^c^	32.81 ± 0.79^c^	57.16 ± 1.17^b^
DCS-3	0.046 ± 0.002^a^	17 ± 0.57^c^	42.86 ± 0.79^b^	58.05 ± 0.93^b^
DCS-4	0.045 ± 0.002^a^	16.66 ± 0.26^c^	47.22 ± 0.97^a^	61.59 ± 0.97^a^

#### 3.2.2 WVTR

**Fig. 4:**
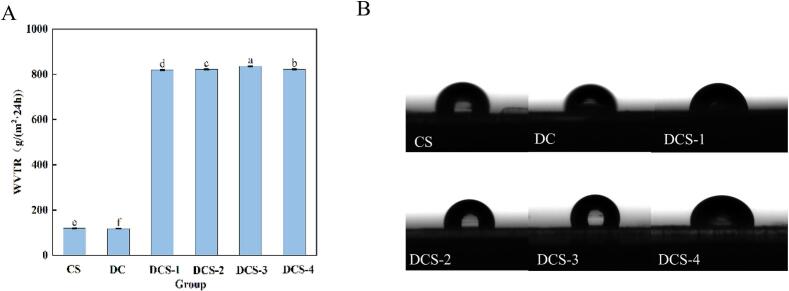
WVTR (A) and WCA (B) of the films.

The ability of food packaging films to prevent moisture loss from food to the surrounding air is crucial. Fig. 4(A) displays the WVTR spectra of CS, DC, DSC-1, DSC-2, DSC-3, and DSC-4 films. WVTR of the DCS composite film was found to be higher owing to the intermolecular cross-linking between SSE and CS. Water vapor permeation behavior of the films occurs via a solution–diffusion mechanism (Gao et al., 2024). The infiltration of H_2_O molecules into CS chains reduces the probability of intermolecular interactions among CS macromolecules. This process facilitates the diffusion of water vapor, thereby accounting for the elevated WVTR observed in films plasticized with SSE and SeP. Besides, an increase in WVTR was noted when the SeP concentration increased. This could be due to the tendency of SeP at high concentrations to aggregate within the film. It is well established that hydrophilic plasticizers, such as glycerol, can enhance the moisture barrier characteristic of hydrocolloid-based films. Similarly, selenium peptides and SSE, as hydrophilic substances, increase water vapor permeability by enhancing hydrophilicity and altering the compactness of the polymer chain structure (Al-Hassan & Norziah, 2012).

#### 3.2.3 CA

A critical metric for determining the hydrophobicity of food packaging materials is the water contact angle (WCA). Firstly, all composite films exhibit hydrophilicity because DES was synthesized from the hydrophilic substances ChCl and citric acid, and SSE and SeP contained a certain amount of hydrophilic groups (Hansen et al., 2021). An increase in the content of SSE and SeP led to a gradual decline in the film's WCA, which indicates an enhancement of its hydrophilic character.

### 3.3 Physical properties

#### 3.3.1 Optical properties

**Fig. 5:**
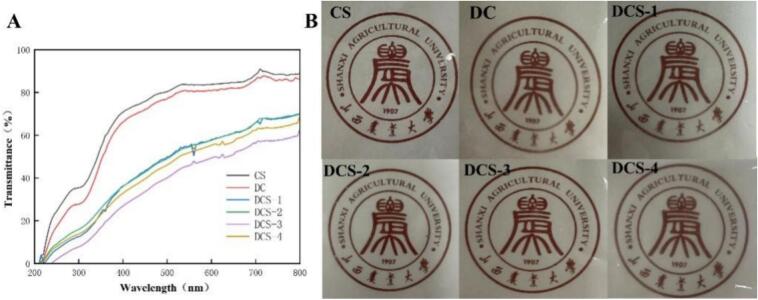
Light transmittance (A) and the images (B) of the films.

The film's ultraviolet (UV) transmittance was measured in order to assess its optical qualities. Because of their colorless transparency and absence of UV-absorbing groups, the CS and DC composite films had significant UV transmittance, as seen in Fig. 5(A). Following the addition of SSE and SeP, the dispersion of SSE and SeP in the DCS composite film decreased the composite film's UV transmittance by either blocking light or creating UV-absorbing groups (Tagrida et al., 2023). The DCS-3 composite film demonstrated exceptionally low UV transmission after increasing the SeP content to 0.25%, suggesting that it possessed superior UV-blocking capabilities. When it comes to fruit packaging, films serve as practical barriers against UV radiation that could cause deterioration during storage and transit.

#### 3.3.2 Mechanical properties

**Fig. 6:**
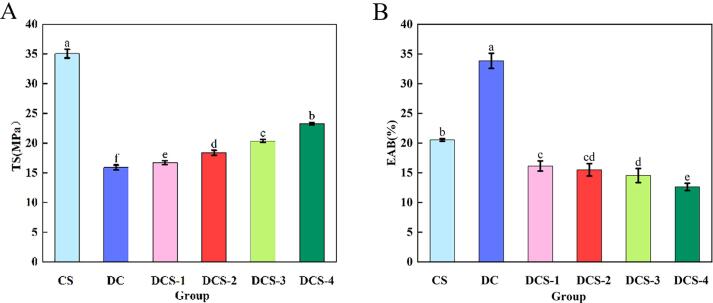
TS (A) and EAB (B) of films.

Films were significantly improved by the added SSE and SeP, as illustrated in Fig. 6. In particular, as the SSE ranged from 0.25% to 0.4%, the films' TS clearly elevated from 16.71 MPa to 18.37 MPa. The addition of SeP was also used to increase the film's EAB, and the maximum EAB of 16.14% was achieved using SSE-0.25%. Besides, as the SeP concentration increased from 0.25% to 0.4%, the tensile performance of DCS films showed an upward trend from 20.34 MPa to 23.27 MPa, while the film's EAB decreased from 14.54% to 12.63%. This is due to SeP's presence of Selenol groups (—SeH), which may resemble hydroxyl groups in terms of electron cloud distribution and reactivity (Pehlivan et al., 2023).

### 3.4 Antioxidant ability

**Fig. 7:**
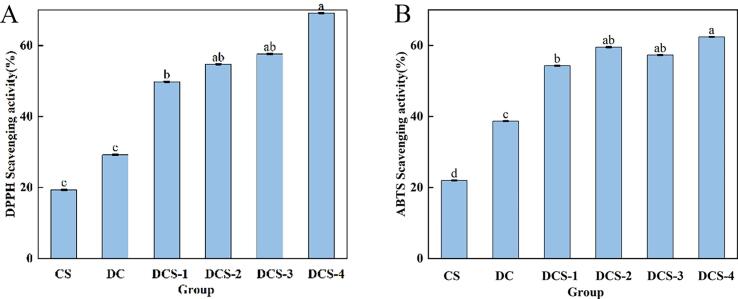
DPPH (A) and ABTS (B) of the films.

Fig. 7 shows the inhibition of ABTS and DPPH radicals by CS films with different SSE and SeP content. Because CS contains amino and hydroxyl groups capable of interrupting ROS-induced oxidation cascades, the effectiveness of pure CS films to suppress DPPH and ABTS radicals was modest but positive. Fig. 7 illustrates how the reductive capacity of DCS strengthened with increasing SSE and SeP additions. This rise was correlated with the amount of SSE and SeP added. The composite films' DPPH radical scavenging rate increased to 69.14% as the amount of SeP added. The potent free radical scavenging ability of flavonoids is largely influenced by their phenolic hydroxyl groups. Because the selenol groups within SePs influence the activity of scavenging free radicals. SePs were found to exhibit a significant antioxidant effect resulting from the combined action of SePs (Zhang et al., 2021). As the SSE concentration in the films was elevated, their ABTS radical scavenging efficiency improved, attaining a maximum value of 59.50%. Because of its inherent reducing qualities, SSE may combine with other chemicals, including free radicals, to change them into stable forms. Se—H bonds in SePs exhibit greater susceptibility to oxidation compared with their counterparts. Organic selenium exerts its antioxidant effect in vitro by mimicking the activity of endogenous antioxidant enzymes and scavenging reactive oxygen species. SePs serve as structural analogues to the active site of glutathione peroxidase (GPx). However, further kinetic or enzyme-specific assays would be needed to confirm a true catalytic mechanism.

### 3.5 Antibacterial ability

**Fig. 8:**
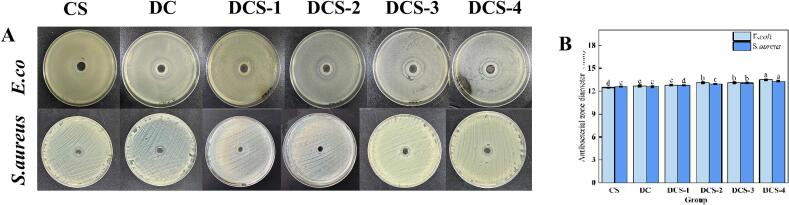
The size of the inhibition zone of the films.

Since bacterial activity is the primary cause of food degradation, active packaging applications greatly benefit from the suppression of food-borne pathogens. As presented in Fig. 8, the composite samples suppressed microbial growth. The CS group demonstrated remarkable antibacterial activity, which resulted from disruption of bacterial membrane integrity by protonated amino functionalities of CS (Nasaj et al., 2024). The addition of DES notably improved the film's antibacterial capacity. Among them, the DCS-4 film displayed the largest zones of inhibition, measuring 13.3 mm against *S. aureus* and 13.5 mm against *E. coli*. The enhanced antibacterial activity of DCS-4 film is likely attributed to the synergistic disruption of bacterial cell membrane integrity by polycationic CS chains and the SeP-induced elevation of intracellular reactive oxygen species (ROS). These ROS overwhelm the bacterial antioxidant defense system, triggering lipid peroxidation and ultimately leading to cell lysis, as evidenced by the large inhibition zones observed(Omardien et al., 2018).

### 3.6 Analysis of the preservation of grapes

**Fig. 9:**
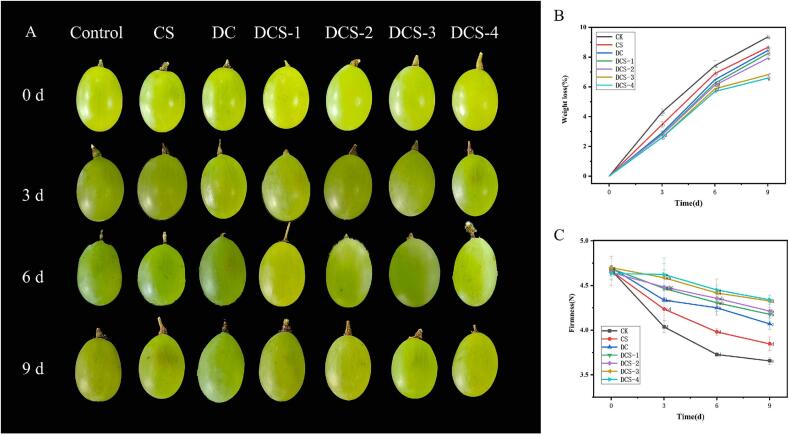
Visual appearance (A), weight loss (B), and firmness (C) of grapes.

**Fig. 10:**
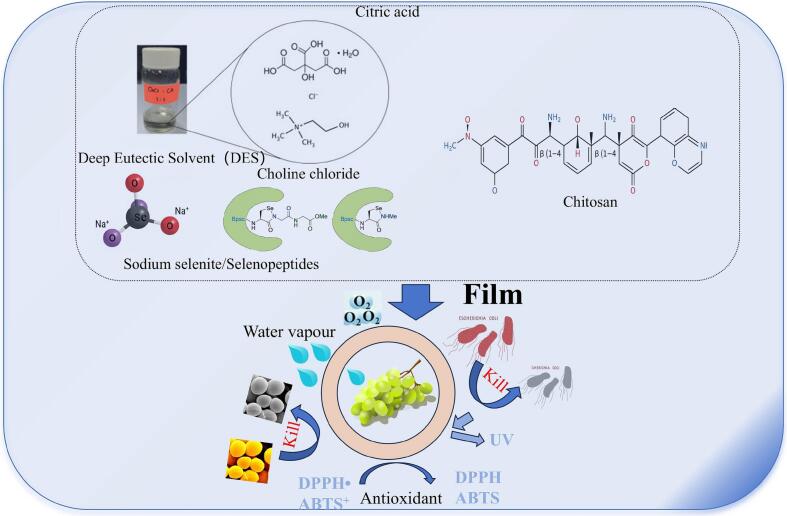
The schematic diagram of film structure and its application in grape preservation.

By analyzing its exceptional mechanical strength, the potential of DCS-4 film for grape preservation was evaluated. After three and six days of storage at 25 °C, respectively, grapes packaged using CS and DC films showed signs of browning, as shown in Fig. 9A. On the other hand, after nine days, grapes wrapped in DCS-3 film only slightly darkened while maintaining their structural integrity. Grape quality was further assessed during storage by measuring firmness (N) and weight loss (%). All groups gradually lost more weight as a result of moisture evaporation, as seen in Fig. 9B. Grapes in DCS film showed a notably lower weight loss (6.58%) after 9 days than those in CS (9.34%) and DC (8.65%) films. Likewise, patterns of firmness were noted (Fig. 9C), with all groups experiencing softening as a result of the action of enzymes that break down cell walls. Relative to fresh grapes (4.67 N), the hardness of the grapes in DC and DCS-4 films decreased to 4.07 N and 4.34 N, respectively, after 9 days. The thiol, diselenide, selenosulfide, amino, hydroxy, and carboxyl groups present in the composite films confer antibacterial activity and free radical scavenging ability, thereby effectively delaying fruit oxidation and spoilage. The reduction in weight loss, despite the high WVTR of the film, is primarily attributable to the formation of a micro-modified atmosphere that suppresses respiration rate and ethylene production, leading to decreased metabolic activity and transpiration. Similar effects have been reported in recent reviews, where optimized various ECF have been reported to inhibit the exchange of CO_2,_ O_2,_ and ethylene for effectively delaying the weight loss in F&V for regulating their extended shelf-life and postharvest quality (Ali et al., 2025). The schematic representation of the film structure and its application in grape preservation is presented in Fig. 10.

## 4 Conclusions

In the present work, we effectively employed the casting approach to create plasticized CS films with SSE and SeP formulations. Se-containing compounds may be able to establish hydrogen bonds with CS and DES, as indicated by FTIR measurement. The majority of the films have a constant section and a flat, homogeneous surface. DCS films have similar mechanical properties to DC films. The physicochemical characteristics of the films exhibited substantial enhancement due to the incorporation of SSE and SeP. The DC films' Young's modulus and TS decreased. As the amount of SSE and SeP grew, so did the DCS film's CA. The film's WS, WVTR, and SD both markedly increased as odium selenite and SeP rose. Additionally, it was noted that DCS films offer outstanding UV-blocking capabilities. Excellent antioxidant activity was demonstrated using DCS-4 film. The study showed that DCS-3 and DCS-4 films possess excellent antibacterial qualities. These suggest that DCS-4 film has potent antioxidant, antibacterial, and UV-blocking qualities. With their superior oxidation, antibacterial, and UV resistance, DCS-4 film is a promising candidate for food preservation packaging. The incorporation of SeP results in films with superior functional properties. Crucially, the substitution of inorganic SSE with SeP significantly enhances the safety profile of the active packaging. SeP exhibits much lower acute toxicity and higher bio-availability than inorganic salts, which offers both sustainability and safety in food packaging applications. However, the various applications it may be utilized for are limited by its great hydrophilicity; hence, future research should concentrate on making the film more water-resistant.

## CRediT authorship contribution statement

**Li Li:** Funding acquisition, Formal analysis, Data curation, Conceptualization. **Shushu Pu:** Conceptualization. **Zhixuan Fan:** Conceptualization. **Yaping Liu:** Resources. **Jianbing Di:** Formal analysis. **Yu Wang:** Funding acquisition, Formal analysis. **Wei Ji:** Funding acquisition.

## Declaration of competing interest

The authors declare that they have no known competing financial interests or personal relationships that could have appeared to influence the work reported in this paper.

## Data Availability

Data will be made available on request.
